# Residential scene classification for gridded population sampling in developing countries using deep convolutional neural networks on satellite imagery

**DOI:** 10.1186/s12942-018-0132-1

**Published:** 2018-05-09

**Authors:** Robert F. Chew, Safaa Amer, Kasey Jones, Jennifer Unangst, James Cajka, Justine Allpress, Mark Bruhn

**Affiliations:** 1 Center for Data Science, RTI International, 3040 East Cornwallis Road, Research Triangle Park, NC USA; 2 Division for Statistical and Data Sciences, RTI International, 3040 East Cornwallis Road, Research Triangle Park, NC USA; 3 Geospatial Science and Technology Program, RTI International, 3040 East Cornwallis Road, Research Triangle Park, NC USA

**Keywords:** Machine learning, Deep learning, Scene classification, Probability based, Complex sample design, Clustering, Remote sensing, GIS

## Abstract

**Background:**

Conducting surveys in low- and middle-income countries is often challenging because many areas lack a complete sampling frame, have outdated census information, or have limited data available for designing and selecting a representative sample. Geosampling is a probability-based, gridded population sampling method that addresses some of these issues by using geographic information system (GIS) tools to create logistically manageable area units for sampling. GIS grid cells are overlaid to partition a country’s existing administrative boundaries into area units that vary in size from 50 m × 50 m to 150 m × 150 m. To avoid sending interviewers to unoccupied areas, researchers manually classify grid cells as “residential” or “nonresidential” through visual inspection of aerial images. “Nonresidential” units are then excluded from sampling and data collection. This process of manually classifying sampling units has drawbacks since it is labor intensive, prone to human error, and creates the need for simplifying assumptions during calculation of design-based sampling weights. In this paper, we discuss the development of a deep learning classification model to predict whether aerial images are residential or nonresidential, thus reducing manual labor and eliminating the need for simplifying assumptions.

**Results:**

On our test sets, the model performs comparable to a human-level baseline in both Nigeria (94.5% accuracy) and Guatemala (96.4% accuracy), and outperforms baseline machine learning models trained on crowdsourced or remote-sensed geospatial features. Additionally, our findings suggest that this approach can work well in new areas with relatively modest amounts of training data.

**Conclusions:**

Gridded population sampling methods like geosampling are becoming increasingly popular in countries with outdated or inaccurate census data because of their timeliness, flexibility, and cost. Using deep learning models directly on satellite images, we provide a novel method for sample frame construction that identifies residential gridded aerial units. In cases where manual classification of satellite images is used to (1) correct for errors in gridded population data sets or (2) classify grids where population estimates are unavailable, this methodology can help reduce annotation burden with comparable quality to human analysts.

## Background

Nationally representative survey samples are needed for studies in low- and middle-income countries to support decision-making in research areas ranging from international development to public health. For a probability-based sample, this requires an updated sampling frame with adequate coverage of the target population. For a face-to-face survey of households, a country’s national census may provide an outdated sampling frame. However, to obtain a statistically efficient probability-based sample of households, an up-to-date roster of households within the sampled area units is necessary. This is often unavailable in low- and middle-income countries, so researchers have traditionally relied on field enumeration of the smallest administrative units or random walk to sample households [1]. In a field enumeration approach, researchers conduct a listing of all households within the sampled areas to construct a sampling frame; the sample of households is then randomly selected from this list. A full listing is time consuming and expensive and requires skilled personnel [2], and it is susceptible to main-street bias (oversampling of highly populous areas), among other errors. In a random-walk approach (also called random route sampling), field staff do not enumerate all households within a selected area; instead, they are provided a starting point and a set of instructions for selecting households while in the field (e.g., sample every fourth house along a specified route). This approach is less resource intensive but lacks statistical rigor because of underlying assumptions about the selection method [3, 4], and may be prone to bias because of the effects of interviewer behavior [5–13].

### Geosampling for gridded population sampling

**Fig. 1:**
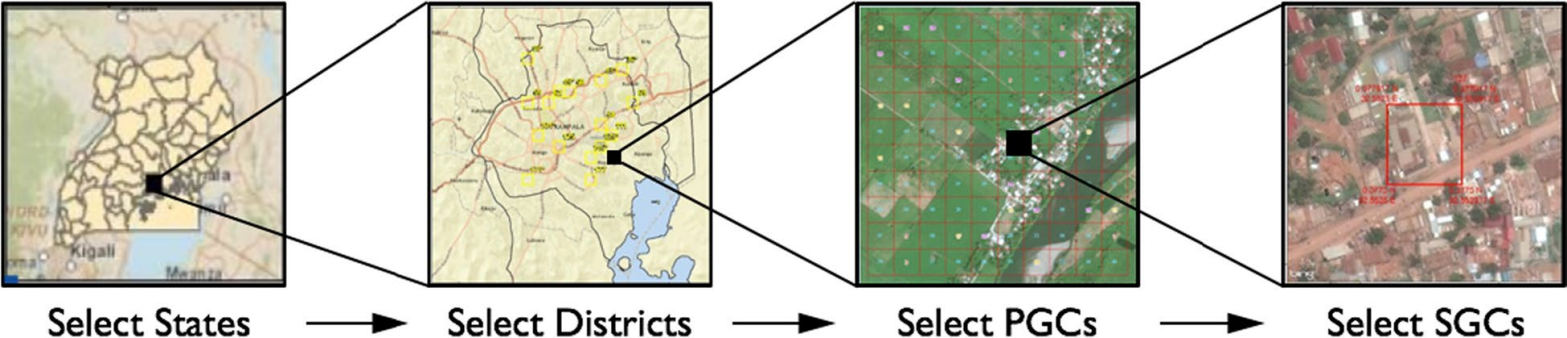
Overview of an example multistage geosampling design outside of Kampala, Uganda

Researchers are developing new and innovative methods that facilitate probability-based survey samples in developing countries at a reasonable cost. One such method, geosampling, uses a geographic information system (GIS) to partition areas of interest into logistically manageable grid cells for sampling [14], contributing to the growing literature on gridded population sampling [2, 15–22]. The first step of geosampling is typically to use a country’s administrative geography (e.g., states, districts) from the most recent census to design a multistage probability-based sample up to the smallest administrative unit with reliable information (Fig. 1). Once the smallest available administrative units are sampled, a grid is overlaid on the sampled units to partition them into 1 km^2^ grid cells, called primary grid cells (PGCs). A probability-based sample of PGCs is then selected with the option of integrating population estimate data, derived from GIS resources such as LandScan [23] into the sample design.

The PGCs are further divided into smaller area units called secondary grid cells (SGCs) using a similar approach, albeit without a population estimate at that lower level, and a probability-based sample of SGCs is selected. Using SGCs as the smallest area unit ensures a manageable area size for the field staff conducting data collection, and reduces the degree to which survey respondents are clustered in a particular geographic area. High clustering of sampled units can lead to inflated variance estimates, thus reducing accuracy of survey estimates [24]. Interviewers are then instructed to survey all households within selected SGCs, reducing the potential for interviewer selection bias. Note that SGCs can vary in size (50 m × 50 m to 150 m × 150 m grid cells) based on population density and urbanicity, rendering smaller areas in dense urban environments and larger areas in more rural settings. This flexibility in grid size variation is designed to help field staff better manage logistics, as large grid areas in population-dense environments make it more difficult for interviewers to effectively scan the entire targeted area, identify households to include in the sample, and attempt to interview all targeted respondents within the grid unit during one visit.

Given the logistical challenges, it is undesirable and costly to send field staff to uninhabited or sparsely populated areas. Prior to sampling PGCs and SGCs, several steps are taken to refine the set of grid cells eligible for selection. First, PGCs with a LandScan population estimate lower than 250 people per km^2^ are excluded from sampling. While gridded population datasets are becoming more detailed [25], population predictions at smaller area sizes have historically been less accurate than at larger geographic units. In particular, case studies have reported large absolute differences existing across gridded population data sets in more populous regions when compared to low density areas [26], and root mean squared error (RMSE) between gridded population estimates and high spatial resolution population census data increasing as the geographic units are more granular [27]. To help mitigate these inaccuracies, a random sample of PGCs with an estimated population greater than 250 people per km^2^ is selected for visual residential screening. Screening utilizes a human coder who determines if a PGC is residential by using aerial photography to establish the context in which buildings are located. This enables the coder to perceive the likely purpose for the structures. The presentation of residential buildings on an aerial photograph is not uniform within or between communities and countries. It is necessary to consider various geospatial characteristics such as community size, building pattern, and proximity to other land uses when determining whether a building is residential. The final sample of PGCs is selected among those classified as residential.^1^

For SGCs, it becomes difficult to reproduce the screening strategy used for PGCs because LandScan population estimates are not available for SGCs and because the set of SGCs is much larger, increasing the time and cost of screening. Prior applications of geosampling have relied on sequential sampling from a hypergeometric distribution to implement a manageable form of residential screenings for SGCs. A hypergeometric distribution provides the number of successes in sample draws, without replacement, from a finite population of size that contains an exact number of successes (i.e., achieving the draw with the targeted characteristic—in our case, a residential SGC), wherein each draw is either a success or a failure. SGCs are sequentially selected at random, screened for residences, and only enter the sample if deemed residential; this process continues until the desired SGC sample size has been achieved. Because screening ceases before all SGCs within a PGC have been screened, this approach does not provide all the necessary information to calculate appropriate probabilities of selection for residential SGCs. Consequently, a simplifying assumption that the population is uniformly distributed across all SGCs within a PGC must be made during weighting.

### Motivation

Our goal is to create a protocol for how to efficiently and accurately classify SGCs as residential versus nonresidential so that nonresidential grids can be excluded from sampling and accounted for in probabilities of selection. This study assesses the utility of machine learning for this task, as an alternative to manual screening. The advantages of this approach are a reduced level of effort and the ability to create a complete residential screening of all SGCs within sampled PGCs. Furthermore, the availability of complete screening information for SGCs would eliminate the need for simplifying assumptions during calculation of SGC sampling weights. Although geosampling and other methods use satellite imagery for final-stage selection [2, 15–18, 20, 22, 28, 29], this is the first instance, to the authors’ knowledge, of using machine learning to aid in sample frame construction in GIS-enabled sampling methodologies.

### Methods for classifying satellite imagery

The remote sensing community has a long history of detecting geospatial features of interest in satellite imagery. Traditional approaches for feature extraction use spectral properties from individual pixels to determine land use or coverage categories [30–32]. With the wider availability of high-resolution satellite imagery, researchers have expanded to Geographic Object-based Image Analysis (GEOBIA) methods [33–35]. These methods are aimed at identifying and demarcating specific objects of interest, such as lakes or buildings, instead of assigning broad land-cover categories to pixels, such as “water” or “urban.”

Increasingly, deep learning models [36] are being used to analyze satellite imagery on diverse tasks, such as semantic segmentation [37], per-pixel classification [38], and poverty mapping [39]. Deep learning has also been particularly successful in scene classification tasks [40–44], which assign an entire aerial image into one of several distinct land-use or land-cover categories. Conceptually, scene classification is equivalent to a binary or multiclass object recognition task in the computer vision literature, except that input images are aerial landscapes instead of portrait or in-profile photographs. As such, our problem can be framed as a two-category scene classification task (predicting whether a satellite image scene is residential vs. nonresidential), where the model results are used to determine which areas are eligible for the survey selection process.

In the following sections, we discuss components of the study, including the data used to test the approach, the machine learning models used for scene classification, and the results. We assess the performance of our deep convolutional neural network (CNN) models against two benchmarks: (1) a human baseline representing the raw agreement between two independent coders, and (2) a machine learning baseline trained on a set of crowdsourced geospatial features from OpenStreetMaps (OSM) and remotely sensed features from the European Space Agency (ESA) Land Cover data set. To better understand the generalizability and reproducibility of our approach, we have tested the models in two different countries—Nigeria and Guatemala—and evaluated the extent to which model accuracy is affected by changes in the training set sample size. Lastly, we conclude with discussions on the approach and future work.

## Methods

### Data preparation

The data used for this study are from two geosampling-based projects. The first data set is from a random subsample of SGCs from the states of Lagos and Kaduna in Nigeria. All SGCs in the subsample were manually screened and then split to create training and test data sets. An additional data set, which included SGCs from Guatemala City, was used to validate the model’s generalizability across different countries and geographic settings. The process of generating the SGC images was the same for both Nigeria and Guatemala. Table 1 summarizes the different grid areas sizes for the Nigeria and Guatemala data sets, respectively.

**Table 1:** Count of images by grid area sizes in the Nigeria and Guatemala data sets

Type	Size	Nigeria	Guatemala
PGC	1 km × 1 km	71	6
SGC	50 m × 50 m	410	1200
SGC	100 m × 100 m	3900	300
SGC	150 m × 150 m	1044	0

Aerial and satellite images were retrieved through three web map services, providing global access to recent Google, Bing, and Esri base maps and imagery. Grid-based polygon layers for both PGCs and SGCs were constructed in ArcGIS, and the source of the imagery at the time of the survey was recorded for future reproducibility. Google and Bing image services are commercially available to ArcGIS users for a modest license fee, and Esri imagery is natively integrated into the GIS software. While these tiled image services provide worldwide coverage, they can vary in both age and spatial resolution from 1 to 2 m depending on the specific geographic location. As such, the imagery provided by each of these services may differ in resolution, color balance, brightness, and cloud cover from location to location, and between vendors. To help determine the best imagery for identifying residential areas for a given location, a graphical user interface (GUI) was developed to help human coders toggle between the different imagery services while classifying grids as residential versus nonresidential (see “Gold-standard labels” section for more detail on developing gold-standard labels). Although using different imagery sources complicates the analysis, it exposes the methodology to implementation scenarios that research teams may realistically encounter. Model performance across imagery sources and grid area sizes are presented in the Results.

**Fig. 2:**
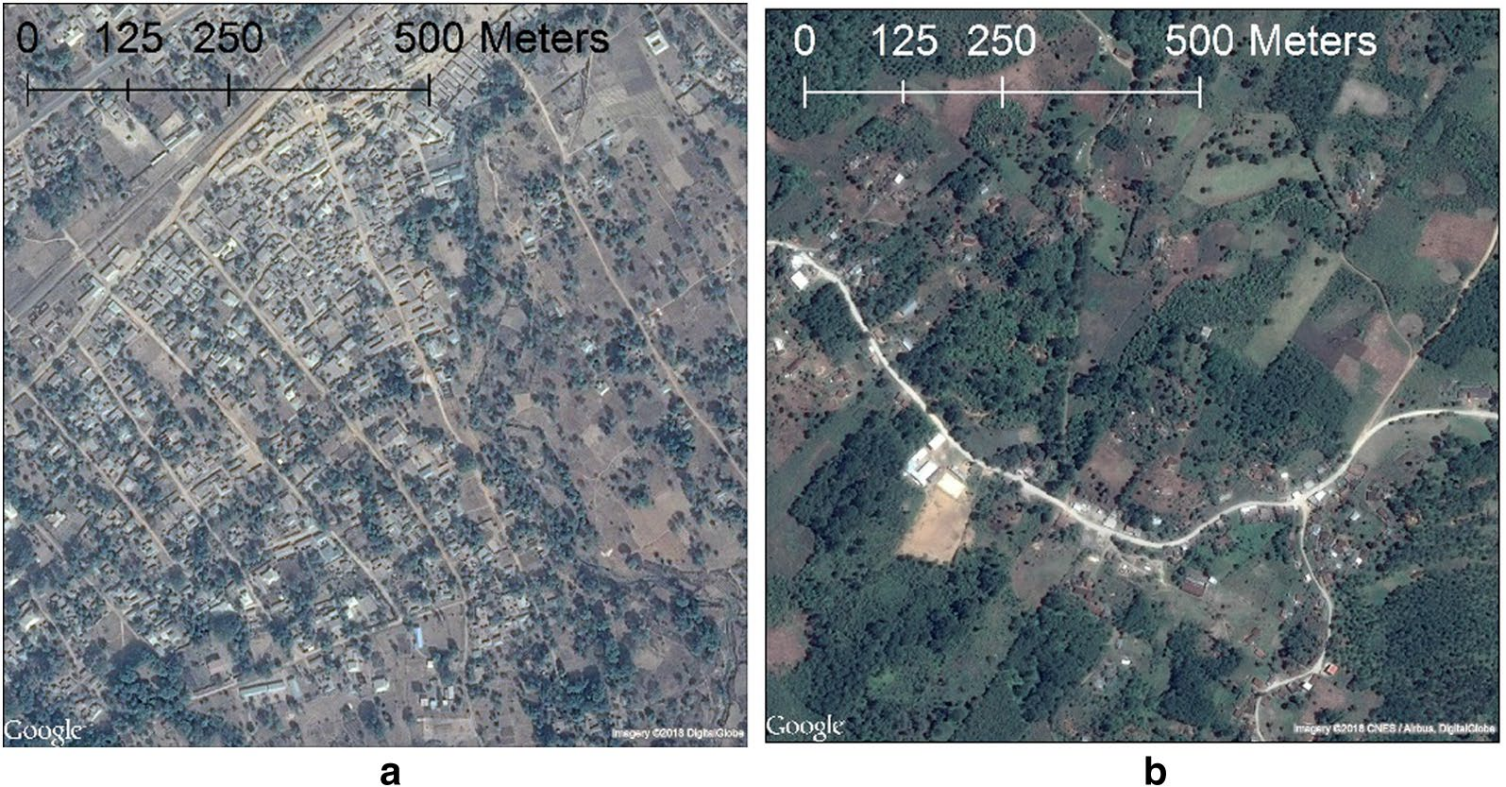
**a** Nigeria PGC Image (1 km × 1 km). **b** Guatemala PGC Image (1 km × 1 km)

We selected 71 random PGCs in Nigeria that contained residential development (Fig. 2a), as well as an additional 6 PGCs in Guatemala (Fig. 2b). Because of the relatively smaller sample size in Guatemala, diversity in urbanicity and geographical characteristics were considered for PGC selection instead of a purely random selection to ensure better generalizability.

From these PGCs, 5350 SGC images were created for Nigeria and 1500 for Guatemala. The size of the secondary grid unit was determined by its level of urbanicity as defined from the latest country census. Urban areas had smaller grid cells than rural areas to account for population density, to avoid high clustering, and so that field staff would have a more consistent workload across SGCs.

**Fig. 3:**
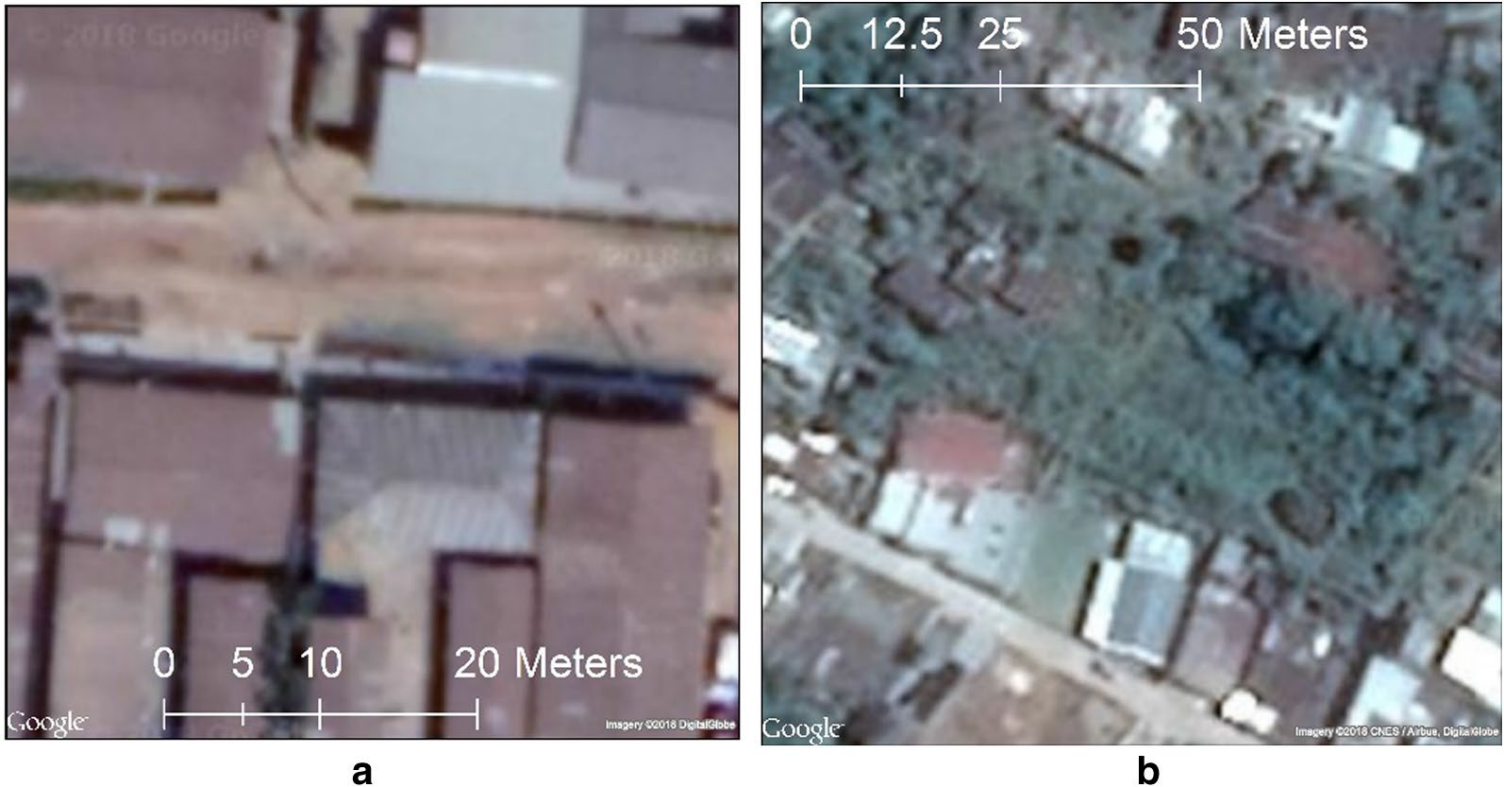
**a** Nigeria SGC Image (50 m × 50 m). **b** Guatemala SGC Image (100 m × 100 m)

Although this adds complexity to the modeling task, we included it in the study to more realistically mirror survey field work considerations. The Nigerian images were composed of 410 grid cells of 50 m × 50 m, 3896 grid cells of 100 m × 100 m, and 1044 grid cells of 150 m × 150 m images. The Guatemalan set was composed of 1200 grid cells of 50 m × 50 m and 300 grid cells of 100 m × 100 m images. Figure 3a shows an example SGC grid in Nigeria and Fig. 3b shows an example SGC grid in Guatemala.

### Labelling data

#### Gold-standard labels

To develop the gold-standard labels of whether a grid is considered “residential” or “nonresidential,” SGCs were individually evaluated by coders to determine if they contained one or more buildings within the image. If the image contained one or more buildings, the entire grid was considered “residential”; otherwise, it was considered “nonresidential.” Since there is a certain amount of subjective decision making required by the coders to determine if buildings are present, the data were labelled by two independent coders, with a senior GIS analyst acting as an adjudicator to settle disputed labels and to ensure consistency and accuracy in selection. The instances of coder disagreement were the motivation for the human benchmark metric (“Human benchmark metric” section) and is further examined in the “Discussion” section.

**Fig. 4:**
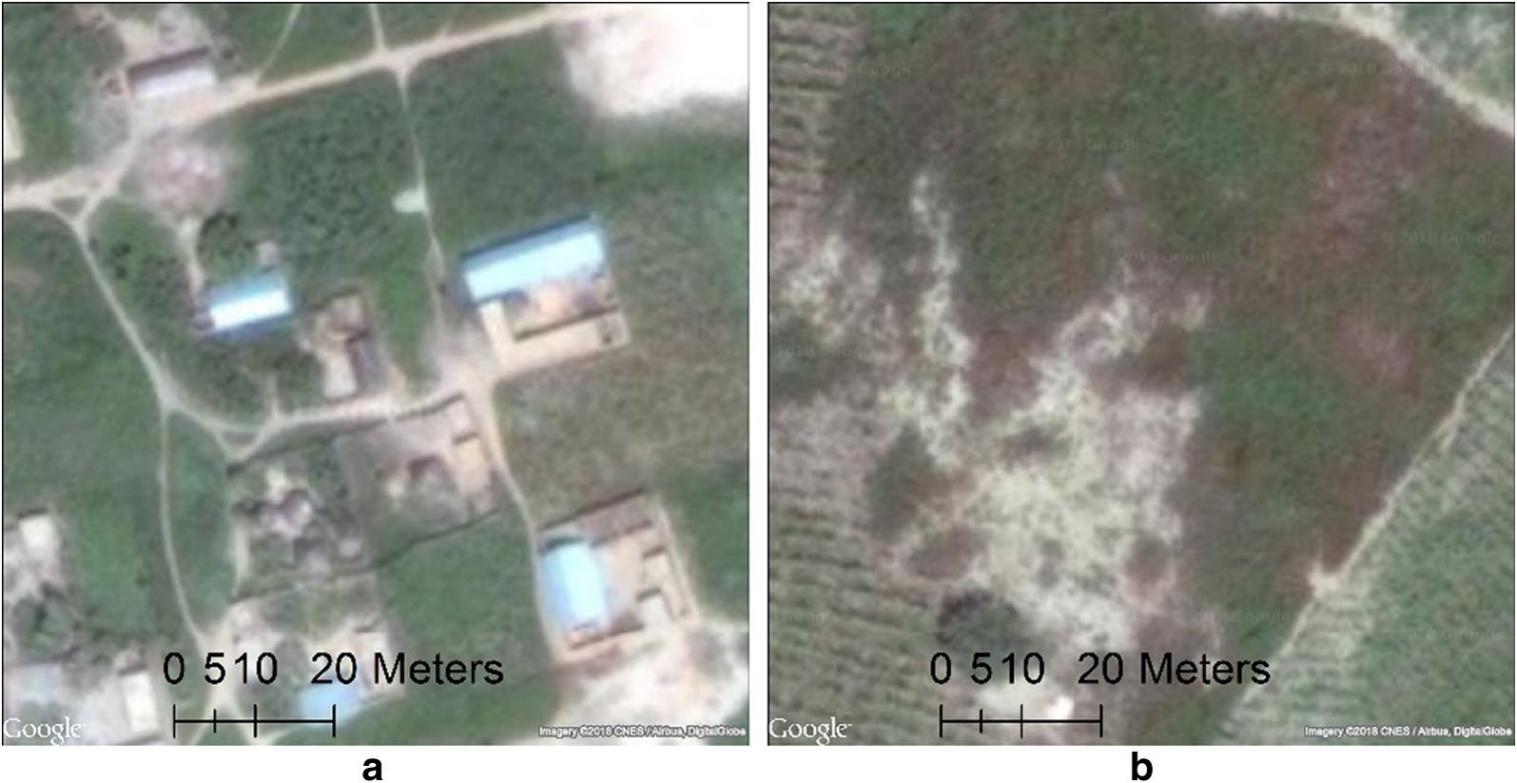
**a** Example SGC residential scene (100 m × 100 m). **b** Example SGC nonresidential scene (100 m × 100 m)

This process was completed using a GUI tool developed within ArcGIS for applying a residential or nonresidential label to each of the grid cells. Figure 4a provides an example residential SGC image whereas Fig. 4b presents an example nonresidential image.

#### Human benchmark metric

To provide a naïve human-level benchmark for how consistently coders agree on labels for this task, we computed the raw agreement [45] between our two independent coders, prior to adjudication. The raw agreement for two coders can be calculated using the following formula:

$$Raw\,Agreement = \frac{1}{N} \mathop \sum \limits_{i = 1}^{C} n_{ii}$$

where *N* is the total number of images that are jointly labelled by the two coders, *n*_*ij*_ is the number of cases assigned as *i* by Coder 1 and *j* by Coder 2 for categories *i*, *j* = 1, …, *C* and *C* is the total number of categories (in our case, residential and nonresidential).

Although other measures of inter-rater reliability have been developed to correct for when coder agreement occurs by chance [46, 47], there are several benefits to using raw agreement for comparison. First, it provides the cleanest comparison to classification model predictions, because it is mathematically equivalent to the “overall accuracy” evaluation metric commonly used in scene classification tasks. The only distinction between the two is that raw agreement compares the difference in labels between two humans, whereas classification accuracy typically compares the difference between a gold-standard human label and a model prediction. Second, inter-rater reliability measures that account for agreement that is expected to occur through chance, such as Cohen’s kappa, can be controversial depending on the context. In the social and health sciences, Cohen’s kappa has been criticized for (1) its “base rate problem” [48], the difficulty in comparing kappa statistics across studies due to the statistic’s dependence on the proportions of positive and negative examples in any given sample, and (2) the assumptions the statistic inherently makes about the decision-making process of raters, which should instead be explicitly modeled for each rater individually [49]. In the remote sensing community, the kappa statistic has been heavily criticized for its use in assessing land change, being scrutinized for reasons such as its assumption of randomness being an irrelevant baseline for many spatial classification problems [50] and being redundant, since it is highly correlated with overall accuracy [51]. For these reasons, raw agreement was used in this study over other reliability metrics, although additional evaluation measures were used to assess model performance (“Model evaluation” section).

Of the 5350 Nigerian images, coders disagreed on labels for 482 grids, resulting in a raw agreement of 91.0%. Of the 1500 Guatemalan images, coders disagreed on 44 grids, resulting in a raw agreement of 97.1%.

### Training and test sets

The Nigeria and Guatemala data were randomly split into training sets for building models (85%) and test sets for model evaluation (15%), stratified to preserve the class ratios of residential and nonresidential images found in the overall data. Although not severely unbalanced, nonresidential grids were more common than residential grids in both our Nigeria (63/37) and Guatemala (67/33) samples. Table 2 provides a breakdown of the training and test sets, respectively, by country and class type.

**Table 2:** Training and test data set allocation for Nigeria and Guatemala

	Nigeria	Guatemala
Training set	4550	1275
Residential	1676	417
Nonresidential	2874	858
Test set	800	225
Residential	295	73
Nonresidential	505	152
Total	5350	1500

As of writing, most open source machine learning libraries do not support modeling.tiff files, so the images were converted to.png format. Additionally, the images were rescaled from 720p × 720p to 150p × 150p for computational efficiencies, as smaller images allow for faster model training and easier handling of large batch sizes. When applicable, we performed additional pre-processing steps for the pre-trained models assessed for transfer learning, as specified in the original papers [52, 53]. These steps are necessary to ensure that the models produce reliable output by matching the input data format used to originally train the models.

### Residential scene classification models

To create a model that can accurately discern between residential and nonresidential aerial images, we develop a series of scene classification models based on machine learning methods. Machine learning is a subdiscipline of artificial intelligence that focuses on the ability of computers to “learn” how to perform tasks without being explicitly programmed how to do so. For example, rather than hand-code software routines with specific instructions on how to identify residential scenes from images, a model is “trained” to learn how to distinguish between residential and nonresidential scenes from examples of labelled data. Exploring modern machine learning methods for aerial scene classification is attractive due to the near human-level performance they have achieved in tasks as diverse as object recognition [54–56], speech recognition [57, 58], and gaming [59–61]. Additionally, after a model is trained, predicting the classes of new images can be automated without additional human intervention and performed at scale. For the use case of screening grids for residential or nonresidential scenes, these models can be used as the sole screening tool or as an additional quality check to assist a team of human annotators.

**Fig. 5:**
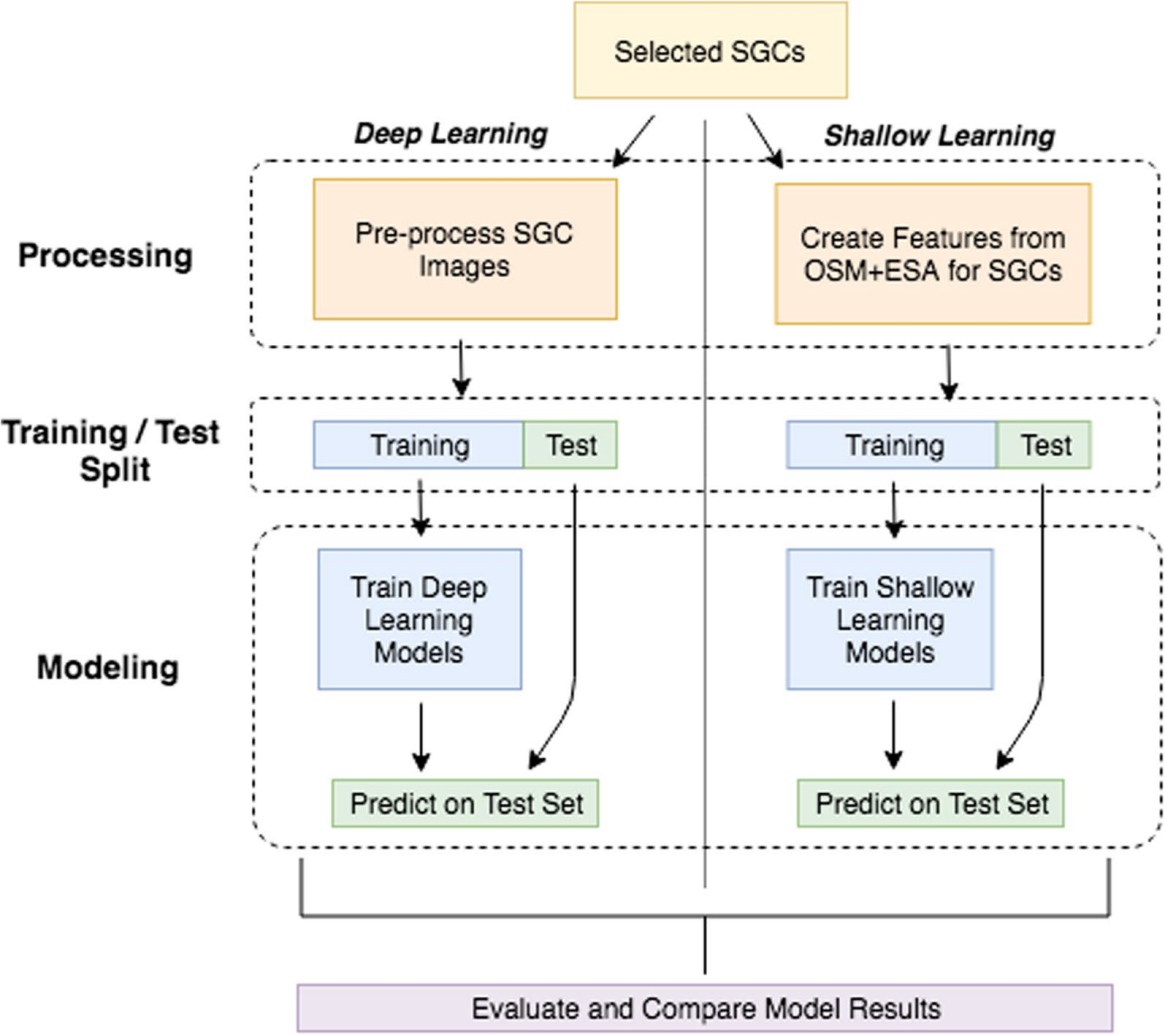
Workflow diagram of modeling approach

The scene classification models presented in this paper can be classified into two overarching groups: (1) “deep learning” models [36], which learn data representations by processing raw data inputs through multiple successive model layers that detect features (most commonly, performed with artificial neural network models) and (2) more traditional “shallow learning” models that learn decision rules from variables (i.e., features) created by modelers with expertise or experience with the phenomena being modeled. In our case, we develop deep learning scene classification models directly from labelled satellite images without explicitly creating variables that distinguish between residential and nonresidential grids. These models are described in the “Deep learning models” section. For comparison, we also develop shallow learning scene classification models with analyst-derived features from the open global GIS datasets OSM [86] and the European Scape Agencies Climate Change Initiative project [62]. These models are described further in the “Shallow learning models” section. A workflow diagram of the two sets of modeling approaches is also included in Fig. 5. In total, there are 11 models developed for Nigeria and another 11 models for Guatemala, whose predictions are compared with each other on the test sets and to the human coder raw agreement scores. Testing such a large number of models is motivated by the No Free Lunch Theorem [63], which states that there are no theoretical guarantees that any one standard machine learning algorithm will work best on all tasks, implicitly promoting an empirical approach to model selection for supervised classification problems.

#### Deep learning models

##### Baseline convolutional neural network

As a baseline deep learning model, we constructed an eight-layered convolutional neural network (CNN) consisting of three convolutional, three pooling, and two fully connected layers. A CNN is a type of artificial neural network model that contains a convolution filter as at least one of its layers. In image processing, a convolution filter (or kernel) is a small matrix of values that, when applied to a larger image, can help isolate notable image features (edges, corners, etc.). Convolution filters use the convolution matrix operation to extract features, often convolving the filter across the image in a sliding window to capture local details. While researchers have developed many specialized filters for feature extraction [64, 65], CNN filters are not specified a priori to extract any specific features. Instead, elements of the CNN filter matrix are included as model parameters and derived during the training process, effectively creating custom filters salient for the specific modeling task. Deep CNNs take this a step further by chaining convolution layers together, a process that ideally captures increasingly higher-level and more nuanced representations of the data. This model uses 3 × 3 convolution filters with a stride of 1 to extract data representations.

Other types of layers besides convolutional layers are often included in CNNs to perform complementary actions. Max pooling [66] was performed in three layers with a 2 × 2 filter to reduce the number of parameters and help prevent overfitting. Max pooling is a simple dimension reduction technique in which a portion of a matrix is isolated and the max value of the isolated elements is returned. This simplified representation summarizes characteristics of the earlier layers, helping later layers generalize more broadly rather than learn traits that are specific only to a particular image. In addition, rectified linear units (ReLU) were used for the activation function to speed up training [67]. Activation functions serve the same purpose as link functions for general linear models (GLMs) in the statistics literature [68], providing a way of transforming a linear predictor into a nonlinear response space. ReLUs differ from other popular activation functions like the logistic sigmoid function (commonly used in logistic regression) in that ReLUs return zero at any input values in the negative domain and return the input value itself in the positive domain:

$$f\left( x \right) = x^{ + } = \hbox{max} \left( {0,x} \right)$$

**Fig. 6:**
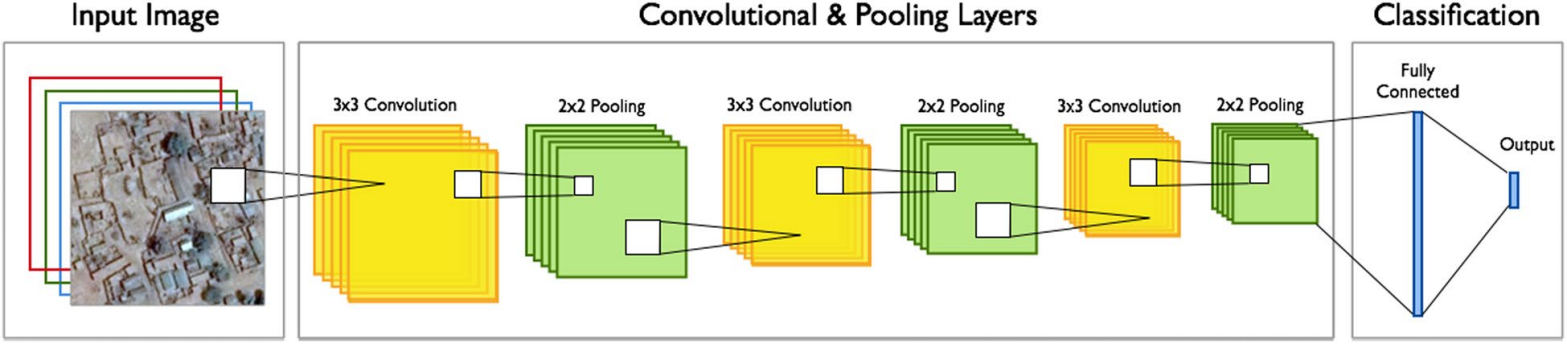
Network diagram of baseline CNN

The first fully connected dense layer also used a ReLU activation function and leveraged a dropout method [p(dropout) = 0.5] to prevent overfitting [69]. Dropout is a regularization technique in which units in your neural network are randomly dropped (along with their connections) during training. The intuition behind this method is that, by thinning the network connections in your fully connected layers, you prevent parameters from being too interdependent among themselves, resulting in a network that will generalize better to new examples. A final dense layer with a sigmoid activation function is used to create predicted probabilities of inclusion for either the “residential” or “nonresidential” classes. The model was run with a batch size of 25 images each and trained over 35 epochs. Figure 6 presents a simplified network diagram of the baseline CNN.

##### Transfer learning

Large labelled data sets or strong regularization are often required to effectively train deep learning models without overfitting [69]. While many state-of-the-art deep learning models have dozens of layers [52, 53], this can results in thousands or even millions of model parameters to fit. Training an exceedingly deep architecture from scratch with random initializations was prohibitive for our sample size, so we used a transfer learning approach [70–72] to leverage stable weights from deep CNN classification models trained on much larger data sets. Transfer learning is a learning framework in which the objective is to use a model trained in one source domain (or task) to help build a model in a related target domain (or task) without the need for considerable new labelled data [70]. This “knowledge transfer” paradigm, in which general features learned from one task help inform a similar task, has become particularly popular with deep CNNs, as pretrained models built on large labelled datasets are often available through open source code repositories.

To test a transfer learning approach, we used the ImageNet dataset as our source domain and the labelled grid scene images as the target domain. ImageNet is a labelled image data set consisting of over 1.2 million high-resolution images and 1000 categories [73], which were collected from the web and labelled by human coders on Amazon’s Mechanical Turk platform. ImageNet categories are based off the lexical database WordNet, which semantically organizes and groups commonly used words into concept hierarchies [74]. As such, ImageNet does not include aerial images because they are not generally associated with archetypical representations of objects (e.g., a standard image of a building would be more likely to be portrayed in profile or as part of a landscape rather than from an overhead view). In addition, aerial images may contain many distinct objects in the same image whereas ImageNet images do not. Even with this limitation, transfer learning with ImageNet trained models have produced state-of-the-art results on images that do not fit this criteria, such as medical [75] and satellite imagery [76].

While it seems unintuitive that a model built on non-aerial images could help develop a model that identifies residential gridded aerial units, deep CNNs have been shown to benefit from spatial hierarchies of patterns [77] in which earlier layers detect small localized patterns (such as edges), while later layers construct more complex representations (such as shapes) composed of the localized patterns detected in earlier layers. While complex representations at later layers can reduce the performance of transfer learning to new tasks if they are too highly specialized [72], research suggests that transferring features even from dissimilar tasks can be better than using random parameter initializations [72]. In addition, transferability tends to increase as the similarity between tasks increases; [72] as such, we favor using pretrained model weights in this study that were originally trained to solve a task similar to ours (Inception V3 [52] and VGG16 [53] used for object recognition).

To test the viability of transfer learning, we used pretrained models from the well-known Inception V3 [52] and VGG16 [53] architectures. Inception and VGG16 are deep CNN model architectures that won first and second place, respectively, at the ImageNet Large-Scale Visual Recognition Challenge 2014 (ILSVRC 2014) and have been used successfully for transfer learning on tasks diverse as cell nuclei classification on histopathology images [78], human aquatic activities classification on micro-Doppler signatures [79], and fruit detection in orchards [80]. Model parameters (i.e., weights) for the architectures trained on ImageNet were acquired through the Python Keras library implementation [81]. To allow the pretrained weights to update for our modeling task, we performed transfer learning in two steps. First, we ran our training and test images through the pretrained Inception V3 and VGG16 networks on all but the top layers, which often consist of a fully connected layer to flatten the dimensionality and an evaluative fully connected layer with a softmax activation function to provide predicted probabilities for class assignment. The top layers of the pretrained models were not included, because we are not interested in predicting the original ImageNet classes. Second, we used the resulting “bottleneck features” [82] as the base for training our own small fully connected model with our classes of interest (residential vs. nonresidential). Our model includes a fully connected layer with ReLU activation units, a dropout layer with a probability of dropout = 0.5, and a final output layer with a sigmoid activation function to produce class probabilities.

As a final experiment, we created an ensemble model [83–85] of our transfer learning models by averaging each model’s predicted probabilities. The premise behind ensemble learning is that a diverse set of models can achieve better predictive performance than any of the individual constituent models alone.

#### Shallow learning models

Although aerial and satellite images provide a direct way of detecting remote land features, modeling on aerial images is unnecessary if the features of interest are already captured in existing data sets. Large, open geospatial databases, such as OSM [86], provide crowdsourced annotations of roads and buildings for areas worldwide. Furthermore, open data sets of land cover categories, maintained by ESA used to study the effects of climate change [62], provide land use and development patterns. As an additional benchmark, we developed classification models using data derived from OSM and ESA to compare the effectiveness of object recognition models using aerial satellite imagery to classification models using features derived from open geospatial databases. To ensure that the methods could be reproduced in new countries, we only considered data sources that were both open source/freely available and had a global scope.

Table 3 provides a list of variables created for the OSM + ESA data set. These variables were assigned to each PGC and SGC by intersecting the grid cell boundaries and the various contributing geospatial layers using ArcGIS. Building and road features were extracted from OSM while major land-cover variables were assigned to the grid cells from the ESA Climate Change Initiative project. The intersection of buildings to grid cell boundaries was performed twice. The first analysis determined if a grid cell contained *any* building while the second intersection only included buildings that were not classified by OSM as having a non-residential use. Examples of non-residential buildings that were excluded from the intersection include churches, stores, and banks. This variable within the dataset is referred to as semi-filtered as OSM building data is not comprehensively attributed. The classification of grid cells using ESA data assigned each SGC with the land cover classification that intersected the largest proportion of the grid cell.

**Table 3:** GIS derived OSM + ESA variables

Variable name	Type	Number	Description
ContainBuildings	Binary	1	Whether an SGC contains an OSM building polygon
SemiFitBuild	Binary	1	Whether an SGC contains a semi-filtered OSM building polygon
AnyRoad	Binary	1	Whether the SGC intersects any OSM road
ResRoad	Binary	1	Whether the SGC intersects any OSM road labelled residential
ResPlusUnRoad	Binary	1	Whether the SGC intersects any OSM road labelled residential or unlabeled
Glob2015_MajLC	Categorical	38	ESA land-cover categories, ranging from “cropland” to “permanent snow and ice”

We assessed the OSM + ESM data set on seven different classifiers (decision trees, gradient boosting trees, AdaBoost, random forest, logistic regression, support vector machines, and k-nearest neighbors) using the scikit-learn package in Python [87]. The models were run for both Nigeria and Guatemala using the same training and test splits as the deep CNN models for comparability.

**Table 4:** Model evaluation metrics for the Nigeria and Guatemala test sets

Model	Type	Acc.	Prec.	Recall	F1
Nigeria					
Baseline CNN	Deep	88.9%	89.2%	88.9%	89.0%
VGG16 with ImageNet weights	Deep	93.4%	93.4%	93.4%	93.3%
InceptionV3 with ImageNet weights	Deep	93.6%	93.6%	93.6%	93.6%
VGG16 and InceptionV3 ensemble	Deep	94.5%	94.5%	94.5%	94.5%
Decision Tree	Shallow	80.3%	80.9%	80.3%	78.9%
Gradient Boosting	Shallow	80.3%	80.9%	80.3%	79.0%
AdaBoost	Shallow	80.6%	81.8%	80.6%	79.2%
Random forest	Shallow	80.1%	80.7%	80.1%	78.8%
Logistic regression	Shallow	80.6%	81.8%	80.6%	79.2%
Support vector machine	Shallow	79.9%	81.5%	79.9%	78.1%
K-nearest neighbors	Shallow	75.6%	81.3%	75.6%	71.3%
Human benchmark	Human	91.0%*	–	–	–
Guatemala					
Baseline CNN	Deep	93.3%	93.3%	93.3%	93.3%
VGG16 with ImageNet weights	Deep	96.4%	96.7%	96.4%	96.5%
Inception V3 with ImageNet weights	Deep	95.6%	95.9%	95.6%	95.6%
VGG16 and InceptionV3 ensemble	Deep	96.4%	96.7%	96.4%	96.5%
Decision tree	Shallow	93.8%	94.1%	93.8%	93.8%
Gradient boosting	Shallow	93.8%	94.1%	93.8%	93.8%
AdaBoost	Shallow	92.9%	93.1%	92.9%	93.0%
Random forest	Shallow	93.8%	94.1%	93.8%	93.8%
Logistic regression	Shallow	93.8%	94.1%	93.8%	93.8%
Support vector machine	Shallow	93.8%	94.6%	93.8%	93.9%
K-nearest neighbors	Shallow	92.4%	93.7%	92.4%	92.6%
Human benchmark	Human	97.1%*	–	–	–

*Raw agreement between two independent coders

### Model evaluation

To evaluate model performance on the test set, we used the following four metrics to assess different aspects of the predictions:

1. *Overall accuracy*—percent of correct predictions.
2. *Precision*—true positives/(true positives + false positives). Indicates the number of true positives out of all observations that are predicted positive (i.e., of all the grids that are predicted residential, the percentage that are actually residential).
3. *Recall*—true positives/(true positives + false negatives). Indicates the number of true positives detected (i.e., the percentage of all residential grids predicted residential by the model).
4. *F1*-*score*—harmonic mean of precision and recall:
  $$F_{1} = \frac{2}{{\frac{1}{recall} + \frac{1}{precision}}}$$

These metrics were calculated for each model evaluated, on both data sets. For additional model assessments, we compared overall accuracy across imagery sources (Google, Bing, Esri) and SGC grid area sizes (50 m × 50 m, 100 m × 100 m, 150 m × 150 m). Last, we tested the model sensitivity with respect to the amount new of training data required, to better understand the expected data annotation burden on future survey projects.

## Results

### Scene classification model results

Table 4 presents model evaluation metrics across the model runs for both Nigeria and Guatemala. Raw agreement of the two independent coders is also provided as the human-level benchmark.

Of the four deep learning models assessed (baseline CNN, VGG16, IncetionV3, and VGG16 + Inception), the ensemble of VGG16 and InceptionV3 performed the best in Nigeria, with an accuracy of 94.4% and F1-score of 92.2%. The ensemble also performed the best in Guatemala with a test set accuracy of 96.4% and F1-score of 96.5%. Overall, the transfer learning models performed considerably better than the baseline CNN, with over 93% accuracy for both VGG16 and InceptionV3 in Nigeria (compared to 88.9% for the baseline CNN) and over 95% for both in Guatemala (compared to 93.3% for the baseline CNN). Both the transfer learning models and the ensemble compared favorably to the human benchmark for Nigeria, performing better than the raw agreement (94.5 vs. 91.0%). These models almost performed as well as the human benchmark in Guatemala (96.4 vs. 97.1%).

As a further comparison, we created shallow classification models using GIS-derived variables from OSM and ESA to predict residential grids in Nigeria and Guatemala. Using the same grids for training and test sets as the deep learning models, our best model accuracy using the OSM + ESA variables was 80.6% in Nigeria and 93.8% in Guatemala (Table 4). In Nigeria, all models except k-nearest neighbors performed similarly, with AdaBoost and logistic regression classifiers performing slightly better than others. In Guatemala all models performed in a tight range between 92.4 and 93.8%, although only k-nearest neighbors and AdaBoost achieved an accuracy lower than 93.8%. Precision, recall, and F1-scores were also stable and consistent within country samples.

Compared to the deep learning models trained directly on images, the shallow learning models using OSM + ESA variables performed worse in both Nigeria and Guatemala. Although model accuracy was relatively close between the image and OSM + ESA models in Guatemala (93.8 vs. 96.4%), there was a substantial difference in performance in Nigeria (80.6 vs. 94.4%). In addition, unlike the image-based models, the OSM + ESA models greatly underperformed the human-level benchmark in Nigeria (80.6 vs. 91.0%), while also slightly underperforming in Guatemala (93.8 vs. 97.1%).

A possible explanation for the difference in performance of the OSM + ESA models between Nigeria and Guatemala may be because of the completeness of the OSM database for the two countries. Evidence of this comes from a recent study on road network completeness in OSM [88], which found that Nigeria had a lower estimated fraction of roads captured (36%) than Guatemala (47%). Although using a GIS feature model may become more reliable as developing countries get better coverage, the models trained on satellite images in this study do not suffer from this limitation.

### Effect of imagery source and grid area sizes

As our data sets in Nigeria and Guatemala contain multiple image sources and grid area sizes, we test to see if accuracy on the best performing model is impacted by either sources of variation. Table 5 reports the test set accuracy across different SGC grid sizes. In Nigeria, the model was most accurate predicting 50 m × 50 m grid size images (98.65%), followed by the 150 m × 150 m grid sizes (95.48%). The model was least accurate in predicting the 100 m × 100 m grid size images (93.52%). However, as the accuracies fall in a small range, we performed a 3-sample test of proportions to account for the differences in accuracy that may occur due to chance. The test results do not provide substantial evidence to reject the null hypotheses that all the accuracy measures across SGC grid sizes are equal, given *α* = 0.05 (Chi-square = 3.691; *p*-value = 0.1579). Likewise, while Guatemala also predicted 50 m × 50 m grids (97.21%) more often than 100 m × 100 m grids (93.48%), the differences in accuracy were also not statistically significant at *α* = 0.05 (Chi-square = 0.595; *p*-value = 0.4403).

**Table 5:** Test set accuracy by SGC grid size

SGC size	Nigeria		Guatemala	
	Count	Accuracy	Count	Accuracy
50 × 50 m	74	98.65%	179	97.21%
100 × 100 m	571	93.52%	46	93.48%
150 × 150 m	155	95.48%	0	–
Test for equality of proportions	*χ*^2^ = 3.691, *df* = 2, *p*-value = 0.1579		*χ*^2^ = 0.595, *df* = 1, *p*-value = 0.4403	

Table 6 reports the test set accuracy across different image sources. While three sets of images were provided for analysts to choose from (Google, Bing, and Esri), no images from Esri were selected for coding. In Nigeria, the model predicted near-identical accuracies across image sources (Google = 94.25%; Bing = 94.90%). The 2-sample test of proportions also reflects this, failing to reject the null hypothesis at an *α* = 0.05 (Chi-square = 0.016; *p*-value = 0.8982). Surprisingly, Google was selected for all 50 m × 50 m grids and Bing was chosen for all 100 m × 100 m grids in Guatemala. As such, the differences in accuracy and test statistics are the same as when stratifying by grid size.

**Table 6:** Test set accuracy by image source

Image source	Nigeria		Guatemala	
	Count	Accuracy	Count	Accuracy
Google	643	94.25%	179	97.21%
Bing	157	94.90%	46	93.48%
Test for equality of proportions	*χ*^2^ = 0.016, *df* = 1, *p*-value = 0.8982		*χ*^2^ = 0.595, *df* = 1, *p*-value = 0.4403	

### Effect of training set size

**Fig. 7:**
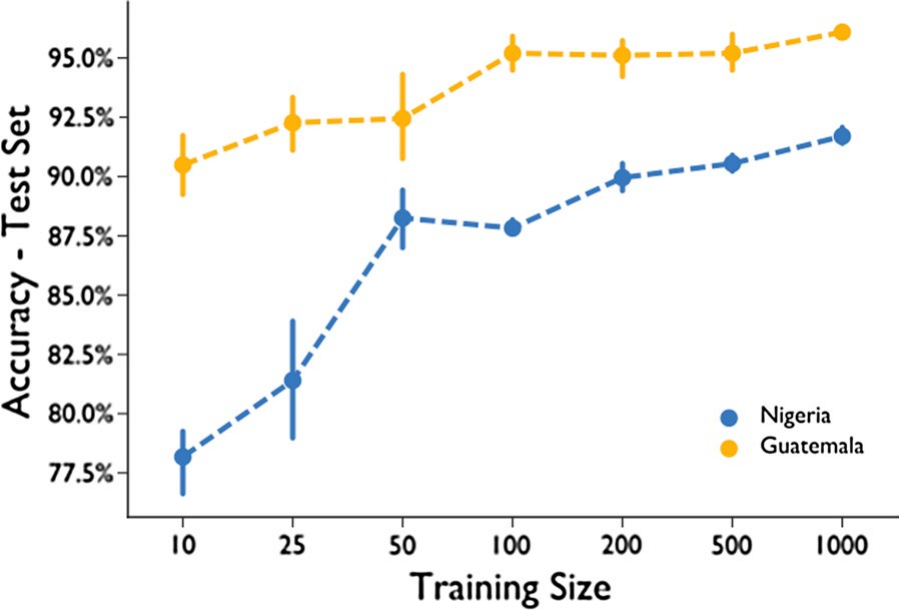
Learning curves for Nigeria and Guatemala

Operationalizing this method in new countries will require retraining the models with images from the new countries. To better understand the expected data annotation burden, we created learning curves to test how sensitive model performance is to training size [89]. Figure 7 shows the test set accuracy and 95% confidence intervals for training set sizes at 10, 25, 50, 100, 250, 500, and 1000 images. Five randomly sampled training sets were created and trained for each set size, stratified to preserve the class ratios seen in the original training sets. The five trained models for each training set size were then run on the corresponding countries complete validation set to determine accuracy metrics. Although results are only presented for the pretrained VGG16 model, the learning curves showed similar trends for InceptionV3.

As expected with smaller training sizes, there is a lower average and larger variance in the accuracy for both Nigeria and Guatemala. Average accuracy increases as training size increases, from 78.2% (n = 10) to 91.7% (n = 1000) in Nigeria and 90.5% (n = 10) to 96.1% (n = 1000) in Guatemala. Although neither sets of models at these sample sizes exceed the human-level benchmarks, they do approach the baseline with modest amounts of training data. This finding both supports the robustness of transfer learning and the more practical case of portability to new areas.

## Discussion

These findings suggest the effectiveness of deep CNNs for identifying residential grids for cluster sampling, providing an accurate and scalable way to help screen large areas with minimal data requirements. Although this method was demonstrated within the context of geosampling, the approach can be applicable to any household survey in low- and middle-income countries with a gridded population sample design. With studies showing a variety of inaccuracies for model-based population data sets at the sub-national level [26, 27], our approach could help verify, supplement, or even replace the need for gridded population estimates in certain cases.

Although little has been published on the use of scene classification for applications in survey research, our results support findings in the remote sensing literature on deep CNNs providing state-of-the-art performance on remote scene classification tasks [90], showing over 95% overall accuracy on data sets containing anywhere from 2 [41] to 45 [91] scene categories. In particular, several studies have also documented the effectiveness of using transfer learning with CNNs pretrained on ImageNet for scene classification tasks [41–43], even though the underlying source data set does not contain satellite images. While other scene classification benchmark datasets [91, 92] can contain up to dozens of different categories (e.g., airplanes, stadiums, beaches, viaduct, etc.), many of these scenes are largely irrelevant for the purpose of household surveys that are only interested in residences. Of comparable studies that publish confusion matrices with scene specific accuracy metrics, residential scenes have been among the most difficult to correctly classify (Table 7). Han et al. [42] and Hu et al. [43], whom both also use a transfer learning approach with deep CNNs pretrained on ImageNet, found that predicted accuracy of residential classes ranged from 85 to 95%, compared with our 94.5% accuracy in Nigeria and 96.4% accuracy in Guatemala. This difficulty in predicting residential scenes may be due to their high similarity to other classes or ambiguity in the definition of what is considered a “residential” scene. Especially when encountering difficult-to-define categories, collapsing classes (such as our overarching “nonresidential” class) can increase classification accuracy by simplifying the modeling task, requiring the model to distinguish only between broad, distinct categories [93]. By focusing on only two scene classes in our modeling, survey researchers not only benefit from a potentially higher accuracy model than if they included additional scenes, but the scenes included are only those relevant for downstream analysis.

**Table 7:** Residential scene classification accuracy across studies using deep CNNs transfer learning models

References	Scene class	Dataset	# Classes	Accuracy (%)	Relative scene accuracy ranking
Hu et al. [43]	Sparse Residential	UC Merced	21	85	19 of 21*
	Med. Residential	UC Merced	21	85	19 of 21*
	Dense Residential	UC Merced	21	90	17 of 21**
Han et al. [42]	Sparse Residential	UC Merced	21	95	12 of 21***
	Med. Residential	UC Merced	21	90	19 of 21****
	Dense Residential	UC Merced	21	85	21 of 21
	Residential	SIRI-WHU	12	93	10 of 12*****
	Residential	WHU-RS	19	88	19 of 19
Chew et al. (in this study)	Residential	Nigeria	2	94.5	NA
	Residential	Guatemala	2	96.4	NA

*Medium and Sparse residential tied for 19th/20th place

**Tied with “intersection” for 17th/18th place

***Tied with seven other classes for 12th–18th place

****Tied with “storage tank” for 19th/20th place

*****Tied with “idle land” for 10th/11th place

In addition to providing survey research teams with a method for screening residential areas, our work also provides contributions to the larger scene classification literature. While deep CNNs have been effective on scene classification tasks ranging in spatial resolutions (2-m resolution in SIRI-WHU dataset [94] to 1-ft resolution in UC Merced dataset) [95], few studies have reported applying deep CNN scene classification models to datasets containing multiple spatial resolutions as found in our data set. We do not find statistically significant differences in accuracies between grid area sizes and image sources, suggesting that deep CNN models can perform well on image datasets that contain heterogeneous properties and that may resemble data collected by survey research and implementation teams on projects in developing countries. Additionally, most other benchmark scene classification datasets contain images from developed areas, such as the United States [40, 95], Europe [91], and urban areas in China [96], rather than low- and middle-income countries. By extending scene classification to Nigeria and Guatemala, we provide additional evidence that methods shown to be effective in developed nations also apply to developing nations where data quality and availability is generally worse.

While initial results are promising, future work could expand the training set to include a larger and more diverse geographic scope to better understand how the method generalizes across developing nations. Furthermore, since SGC images are localized within PGCs, our training samples are highly clustered geographically. This is appropriate for our use case; however, future research could validate if the high accuracy found in this study applies when predicting random SGC grids within a country. Extended analyses could also examine the extent of spatial autocorrelation among residential grids and assess if methods that explicitly model this dependence (e.g., Markov random fields) can help improve model accuracy. In future work, deep learning models could also be applied at the PGC level. Although this could reduce the existing multistep process that is required to implement manual residential screening down to a single step, it is unclear whether the heterogeneity within the larger PGCs would impact the effectiveness of the method.

One limitation of our study was that our nonresidential grids contained a variety of landscapes, including agricultural, forested, and predominately commercial areas without residencies. While we argue that focusing the problem specifically on residential versus nonresidential will likely be preferred for gridded population sampling for household surveys, future research can be directed toward better understanding whether creating more granular scene categories for nonresidential grids can refine the screening process, particularly in helping disambiguate areas in the built environment (residential vs. commercial). This option would need to be balanced against the additional labelling burden of coders needing to choose among multiple classes. The current geosampling methodology only requires knowing whether residential buildings are present in the area. However, the task could be reframed as an object detection problem with the objective of identifying the number of buildings in a grid instead of just the presence or absence of residential buildings. The extension of this work to an object detection task could facilitate the estimation of population estimates for SGCs or may allow direct selection of households from aerial images.

Lastly, although we present these metrics as an assessment of how well our models compare to human performance on this task, we recognize that the specific values for the human-level benchmark are only representative of the coders recruited to assist for this study. Coders with different levels of experience, skill, and conscientiousness than ours would likely produce different results. Additionally, these numbers represent the disagreement across both training and test sets in Nigeria and Guatemala, whereas the model predictions are only assessed on the test sets. Nonetheless, these ballpark figures do provide us greater assurance of this method’s merits compared to the status quo and much needed context to the raw model performance metrics.

## Conclusion

Using deep CNNs, we demonstrated that we can correctly classify whether areas are residential or nonresidential from aerial satellite images, meeting or exceeding a human-level benchmark in both Nigeria and Guatemala. Not only does this capability reduce the manual resources and calendar time needed for labelling images on future geosampling projects, but it will also improve calculation of probabilities of selection at GIS sampling stages by avoiding unnecessary assumptions about the population distribution. Our findings also suggest that this approach can work well in new areas with relatively modest amounts of training data. Lastly, in areas where GIS variables from data sources like OSM are well populated, using GIS derived feature variables can also accurately detect whether a grid is residential or nonresidential. However, our findings suggest that using CNNs trained on satellite images work even when crowdsourced spatial data sets are not well populated or maintained.

## Abbreviations

CNNs: convolutional neural networks
ESA: European Space Agency
GEOBIA: Geographic Object-based Image Analysis
GIS: geographic information systems
GUI: graphical user interface
OSM: OpenStreetMaps
RMSE: root mean squared error
PGC: primary grid cells
ReLU: rectified linear unit
SGC: secondary grid cells

## Footnotes

1 A residential PGC or SGC is a grid cell with at least one building structure detected in the aerial imagery.
